# Dynamic Distribution of HIG2A between the Mitochondria and the Nucleus in Response to Hypoxia and Oxidative Stress

**DOI:** 10.3390/ijms23010389

**Published:** 2021-12-30

**Authors:** Celia Salazar, Miriam Barros, Alvaro A. Elorza, Lina María Ruiz

**Affiliations:** 1 Institute of Biomedical Sciences, Faculty of Health Sciences, Universidad Autónoma de Chile, Santiago 8910060, Chile; celia.salazar@uautonoma.cl; 2 Confocal Microscopy Laboratory, Universidad Andres Bello, Santiago 8370146, Chile; miriam.barros@unab.cl; 3 Institute of Biomedical Sciences, Faculty of Medicine, Universidad Andres Bello, Santiago 8370146, Chile; alvaro.elorza@unab.cl; 4 Institute of Biomedical Sciences, Faculty of Life Sciences, Universidad Andres Bello, Santiago 8370146, Chile; 5 Millennium Institute in Immunology and Immunotherapy, Santiago 8331150, Chile

**Keywords:** *HIGD2A*, hypoxia, oxidative stress, mitochondria, metabolic reprograming, cancer

## Abstract

Mitochondrial respiratory supercomplex formation requires HIG2A protein, which also has been associated with cell proliferation and cell survival under hypoxia. HIG2A protein localizes in mitochondria and nucleus. DNA methylation and mRNA expression of the *HIGD2A* gene show significant alterations in several cancers, suggesting a role for HIG2A in cancer biology. The present work aims to understand the dynamics of the HIG2A subcellular localization under cellular stress. We found that HIG2A protein levels increase under oxidative stress. H_2_O_2_ shifts HIG2A localization to the mitochondria, while rotenone shifts it to the nucleus. HIG2A protein colocalized at a higher level in the nucleus concerning the mitochondrial network under normoxia and hypoxia (2% O_2_). Hypoxia (2% O_2_) significantly increases HIG2A nuclear colocalization in C2C12 cells. In HEK293 cells, chemical hypoxia with CoCl_2_ (>1% O_2_) and FCCP mitochondrial uncoupling, the HIG2A protein decreased its nuclear localization and shifted to the mitochondria. This suggests that the HIG2A distribution pattern between the mitochondria and the nucleus depends on stress and cell type. HIG2A protein expression levels increase under cellular stresses such as hypoxia and oxidative stress. Its dynamic distribution between mitochondria and the nucleus in response to stress factors suggests a new communication system between the mitochondria and the nucleus.

## 1. Introduction

Respiratory supercomplexes represent a regulatory unit of cellular respiration, stabilizing respiratory OXPHOS complexes, enhancing substrate channeling, and minimizing the generation of reactive oxygen species (ROS) during electron transfer reactions [1,2,3,4,5,6,7]. In the last decade, proteins that mediate and regulate the stability and assembly of respiratory supercomplexes have been described, e.g., the SURF1 protein, the MCJ/DnaJC15 co-chaperone, the COX7RP protein, the RCF1 protein, and the HIG2A protein.

In yeast, the RCF1 protein enables the association between complex III and complex IV, promoting the assembly of OXPHOS supercomplexes [8,9,10]. RCF1 is a conserved protein with two orthologs in human mitochondria: HIG1A and HIG2A (in mouse Higd1a and Higd2a). HIG2A is localized in the inner mitochondrial membrane and possesses a hypoxia-inducible domain 2A at the N-terminus [11]. The knockdown of *HIGD2A* in human HeLa cells and mouse C2C12 cells altered the formation of I_1_ + III_2_ + IV_1–4_ supercomplex through the complex IV release [8]. Timón-Gomez et al. (2020) confirmed that HIG2A is required to assemble the COX3 subunit of human complex IV and coordinate supercomplex formation by participating in the association between complex III and IV [11]. HEK293 cells that were knocked-out for the *HIGD2A* gene showed a decrease in mitochondrial respiration, a reduction in the activity of complex III, a decrease in the interaction of complex IV with complex III, a reduction of the biogenesis of complex IV, and a decline in the total levels of complex I [11].

Physiological hypoxia (5% O_2_) increases *HIGD2A* (*Higd2a*) gene expression [12]. Timón-Gómez et al. (2020) showed that HIG2A protein increased in 1% O_2_ hypoxia in the first four- to eight-h during a 48-h exposure [11]; and its overexpression improved cell survival under hypoxia in mammalian models and cell lines, decreasing the levels of hypoxia-induced apoptosis [13].

Analysis of the promoter region of the *HIGD2A* gene indicated the presence of binding sites for transcription factors that were related to cell cycle control, including E2F-1 [12]. The treatment with a selective cyclin-dependent kinases (CDKs) inhibitor, roscovitine, significantly increased *HIGD2A* gene expression in HEK293. In turn, the treatment with the inhibitor of the activation of nuclear transcription factor NF-kappa B, caffeic acid phenethyl ester (CAPE), decreased *Higd2a* gene expression in mouse C2C12 myoblast cells [12]. The inhibition of cell proliferation by CAPE treatment promotes the binding of E2F-1 to the regulatory region of *HIGD2A*, thus establishing a role for E2F-1 in the regulation of *HIGD2A* expression [12].

In HEK293 cells, deletion of *HIGD2A* results in the down-regulation of the NF-kβ pathway following stimulation with Epstein Barr virus latent membrane protein (LMP1) [14]. Also, the silencing the *HIGD2A* gene in C33A human cervical epithelial cancer cells increases the reporter activity of a human papillomavirus oncogene expression, thus suggesting a possible role of HIG2A as a regulator of oncogene expression [15]. The Papillomavirus E2 protein uses multiple cellular proteins to inhibit the expression of its oncogenes and HIG2A could potentially be one of these proteins [15]. Moreover, the silencing of *HIGD2A* alters the viability of DLD1 colon adenocarcinoma cells, indicating that silencing *HIGD2A* induces death in cancer cells, suggesting a role for HIG2A in cell cycle regulation and a potential target in cancer therapy [16]. Remarkably, DNA methylation and mRNA expression in the *HIGD2A* gene showed significant alterations in various cancers [17]. The correlation between high *HIGD2A* expression and poor patient survival is significant for hepatocellular carcinoma, cutaneous melanoma, endometrial carcinoma, and uveal melanoma [17]. All the above-presented studies suggest a role for HIG2A in mitochondrial physiology and cancer biology.

HIG2A was predicted with an importin α-dependent nuclear localization signal. It has a putative motif of DNA binding residues in the alpha-helix [17]. Interestingly, we found that the subcellular localization of the protein HIG2A is to the level of the mitochondrial network and nucleus [12,17]. In the present research, we aimed to understand the dynamics of the HIG2A subcellular localization under cellular stress. For these, we used the HEK293 cell line from human embryonic kidney cells and the C2C12 cell line from mouse C3H muscle myoblast as a study model. HEK293 and C2C12 were selected for their presence of *HIGD2A* and *Higd2A* gene expression, respectively [12,18]. Moreover, *HIGD2A* silencing in HEK293 reduces the assembly and activity of OXPHOS supercomplexes. Also, *Higd2A* knockdown in the C2C12 cells shows alterations in supercomplex assembly [8,11,12]. In addition, in HEK293 and C2C12 the HIG2A protein was observed in the nucleus [12,17]. Our current results show a dynamic distribution of HIG2A between the mitochondria and the nucleus in response to stress factors that may represent a new communication system between the mitochondria and the nucleus.

## 2. Results

### 2.1. Effect of Oxidative Stress on HIG2A Protein Levels in the HEK293 Cell Line

**Figure 1:**
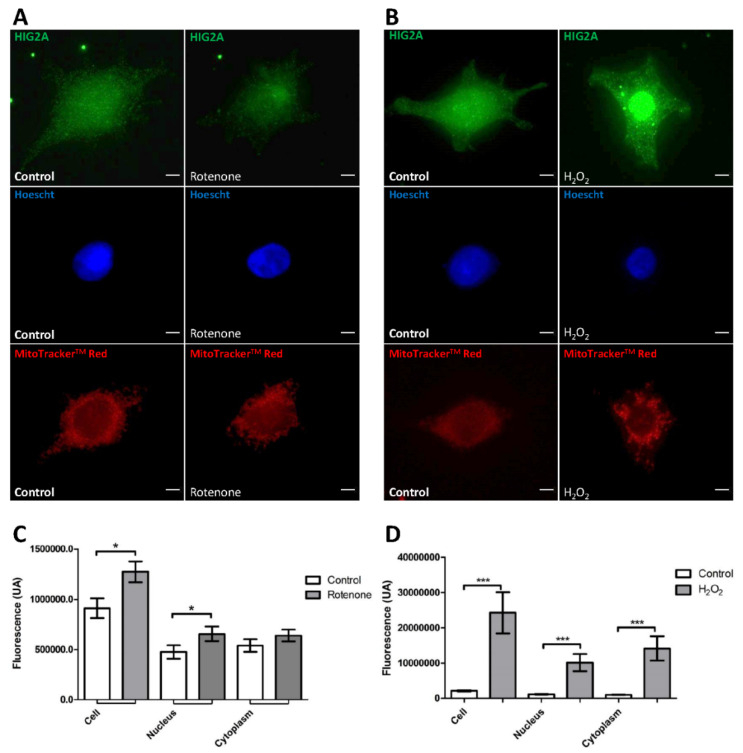
The effect of oxidative stress on HIG2A levels. Representative immunofluorescence images in HEK293 cells; Anti-HIG2A primary antibody, DyLightR 488 secondary antibody (Green). MitoTracker^TM^ Red CM-H2XRos mitochondrial fluorescent marker (Red). Hoescht 33342 (blue signal after binding to DNA). The images were obtained by epifluorescence microscopy (Leica DMI6000). The HEK293 cell line was stressed with 20 nM rotenone for 1-h (**A**,**C**) and with 100 µM hydrogen peroxide (H_2_O_2_) for 1-h (**B**,**D**). The fluorescence of HIG2A in the whole-cell and nucleus was quantified through ImageJ software 1.52p. The fluorescence intensity is represented as an arbitrary unit (AU). Each bar graph represents the mean ± SE, n = 4 biological replicates, number of cells that were analyzed in total (N°): Rotenone N° = 57, Control (rotenone) N° = 22; H_2_O_2_ N° = 98, Control (H_2_O_2_) N° = 67. They were analyzed by a one-tailed *t*-test (*p* ≤ 0.05), followed by a Mann-Whitney test. Statistical differences were found with a significance of *p*-value (*). Bars indicate a 5 µm scale. (* *p*-value ≤ 0.0292; *** *p*-value ≤ 0.0009).

To evaluate the effect of oxidative stress on HIG2A protein levels, we used the HEK293 cell line that was cultured under general and mitochondrial oxidative stress conditions, using H_2_O_2_ and rotenone, respectively. HIG2A levels were analyzed at the cytoplasmic and nuclear levels by immunofluorescence with images acquired using epifluorescence microscopy (Leica DMI6000, Leica Microsystems Inc., Mannheim, Germany) (Figure 1). Rotenone treatment caused a significant increase in HIG2A fluorescence in the nucleus (*, *p*-value 0.0292) (Figure 1A,C). In comparison, the H_2_O_2_ treatment caused a considerable rise in HIG2A fluorescence both in the nucleus (***, *p* = 0.0009) and in the cytoplasm (***, *p* = 0.0009). H_2_O_2_ shifts HIG2A localization to the mitochondria while rotenone shifts it to the nucleus (Figure 1B,D). In addition, the increase in fluorescence is more significant in the presence of H_2_O_2_ concerning rotenone (Figure 1). This assay suggests that HIG2A protein increases when the cells are exposed to oxidative stress (H_2_O_2_ and rotenone). There is a specific cellular distribution depending on the type of oxidative stress in HEK293 cells.

### 2.2. Effect of Hypoxic Stress on the Subcellular Localization of HIG2A in HEK293 and C2C12 Cell Lines

**Figure 2:**
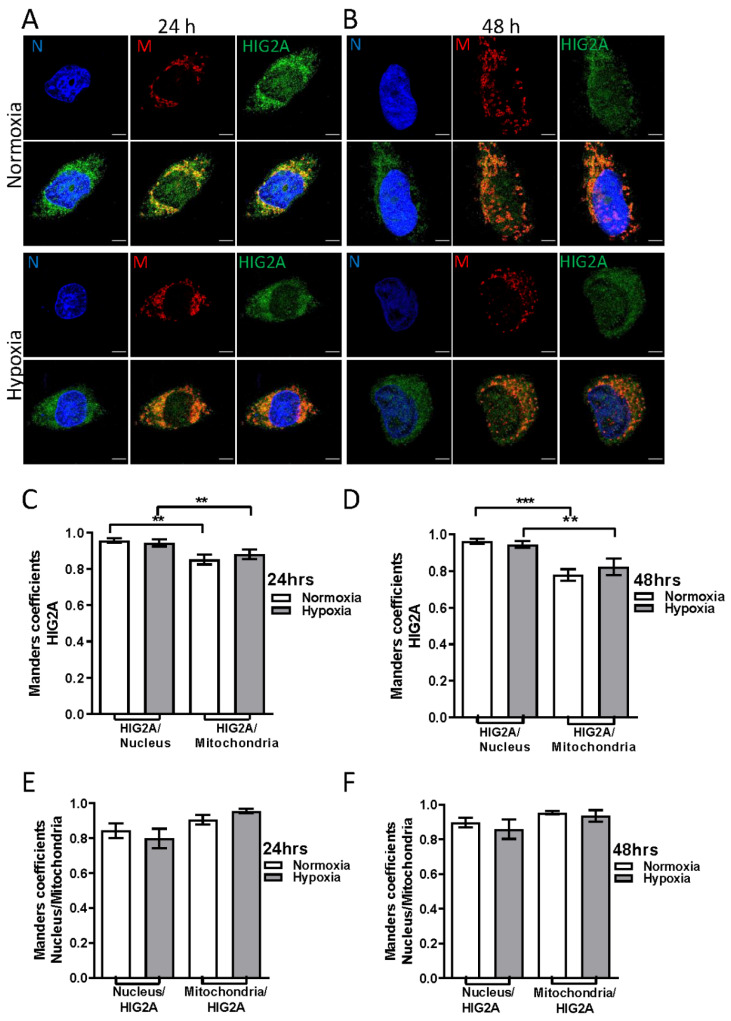
The effect of hypoxia on the subcellular localization of HIG2A in the mitochondria and the nucleus of HEK293 cells. The representative immunofluorescence images in HEK293 cells; anti-HIG2A primary antibody (Abcam ab135399), DyLight^®^ 488 secondary antibody (HIG2A green). MitoTracker^TM^ Red CM-H2XRos mitochondrial fluorescent marker (M red). Hoescht 33342 (blue signal—DNA). Z-axis series were obtained using confocal microscopy (Leica TCS SP8). The separation between each of the slices (Z step size) was 0.130 microns. The HEK293 cells were subjected to hypoxic stress (2% O_2_) for 24-h (**A**,**C**,**E**) and 48-h (**B**,**D**,**F**). The quantification of colocalization was performed by Manders’ coefficient. The fluorescence signal corresponding to HIG2A was quantified over the fluorescence signal of the nucleus (HIG2A/nucleus [M2]) (**C**,**D**) and over the signal of the mitochondria (HIG2A/mitochondria [M1]) (**C**,**D**). Each bar graph represents the mean ± SE, n = 4 biological replicates, 20 cells were analyzed per condition; they were analyzed by a one-tailed *t*-test (*p* < 0.05), followed by a Mann-Whitney test. Statistical differences were found with a significance of *p*-value (** *p* ≤ 0.0011 (**C**), *** *p* ≤ 0.0001 (**D**); ** *p* ≤ 0.0029 (**D**)). ** Very significant (0.001 to 0.01). *** Extremely significant (0.0001 to 0.001). The white bars indicate a 5 µm scale.

**Figure 3:**
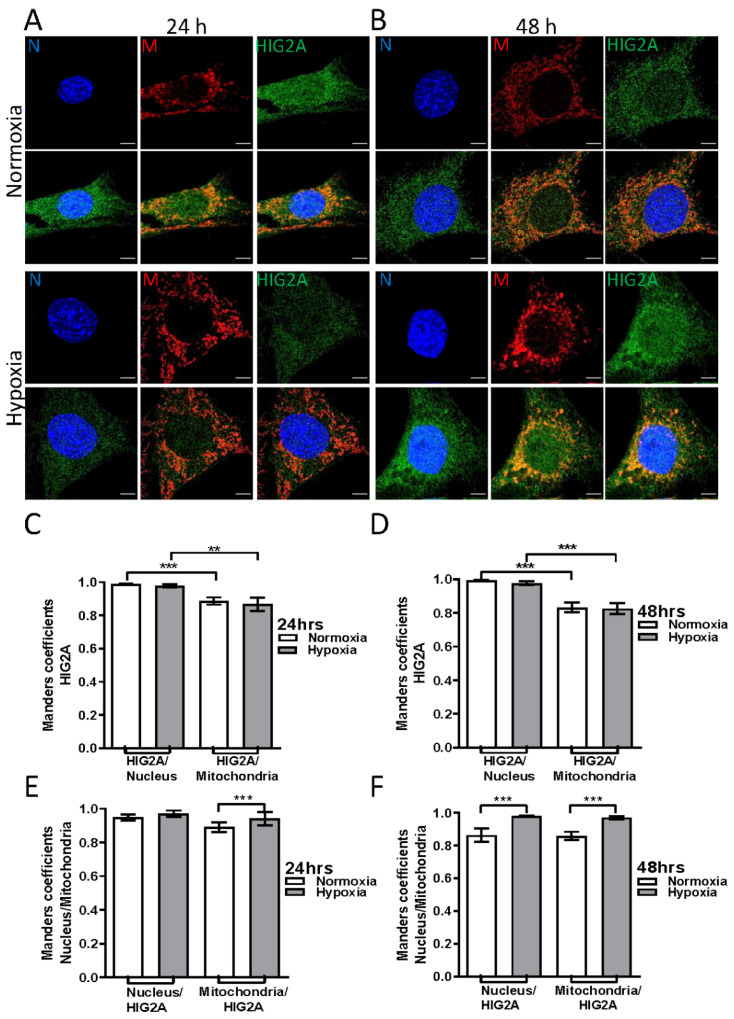
The effect of hypoxia on the subcellular localization of HIG2A in the mitochondria and the nucleus of C2C12 cells. The representative immunofluorescence images in C2C12 cells; anti-HIG2A primary antibody (Abcam ab135399), DyLight^®^ 488 secondary antibody (HIG2A green). MitoTracker^TM^ Red CM-H2XRos mitochondrial fluorescent marker (M red). Hoescht 33342 (blue signal—DNA). Z-axis series were obtained using confocal microscopy (Leica TCS SP8). The separation between each of the slices (Z step size) was 0.130 microns. The C2C12 cells were subjected to hypoxic stress (2% O_2_) for 24-h (**A**,**C**,**E**) and 48-h (**B**,**D**,**F**). The quantification of colocalization was performed using Manders’ coefficient. The fluorescence signal corresponding to HIG2A was quantified over the fluorescence signal of the nucleus (HIG2A/nucleus [M2]) (**C**,**D**) and over the signal of the mitochondria (HIG2A/mitochondria [M1]) (**C**,**D**). Each bar graph represents the mean ± SE, n = 4 biological replicates, 20 cells were analyzed per condition; they were analyzed by a one-tailed *t*-test (*p* < 0.05), followed by a Mann-Whitney test. Statistical differences were found with a significance of *p*-value (*** *p* ≤ 0.0001 (**C**), ** *p* ≤ 0.0073 (**C**), *** *p* ≤ 0.0001 (**D**), *** *p* ≤ 0.0002 (**D**), *** *p* ≤ 0.0002 (**E**), *** *p* ≤ 0.0004 (**F**), *** *p* ≤ 0.0002 (**F**)). ** Very significant (0.001 to 0.01). *** Extremely significant (0.0001 to 0.001). White bars indicate a 5 µm scale.

After hypoxia, C2C12 and HEK293 cells showed a significant increase in the gene expression of *Higd2a* and *HIGD2A*, respectively. In C2C12, this increase was two-fold and three-fold after 36- and 48-h of hypoxia, respectively [12]. We analyzed the colocalization of HIG2A at the mitochondrial and nuclear levels of HEK293 and C2C12 cells that were exposed to hypoxic stress (2% O_2_). We quantified the colocalization of the HIG2A signal (green) overlapping the nucleus signal (blue) (HIG2A/Nucleus [M2]) and the HIG2A signal overlapping the mitochondrial signal (red) (HIG2A/Mitochondria [M1]) (Figure 2C,D and Figure 3C,D). In addition, we quantified colocalization of the nucleus or the mitochondrial signal that overlapped with the HIG2A signal (Nucleus/HIG2A [M1]; Mitochondria/HIG2A [M2]) (Figure 2E,F and Figure 3E,F).

In the HEK293 cells, the HIG2A protein colocalized at a higher level in the nucleus relative to the mitochondrion in both oxygen conditions suggesting that hypoxia (2% O_2_) does not affect HIG2A presence at the nuclear level (Figure 2 and Figure S1, and Table 1).

In C2C12 cells, HIG2A protein colocalized at a higher level in the nucleus relative to the mitochondrion in both oxygen conditions (Figure 3). However, hypoxia (2% O_2_) significantly increased HIG2A colocalization at the nuclear level (Figure 3 and Figure S2, and Table 2).

### 2.3. Effect of Mitochondrial Stress on the Subcellular Localization of HIG2A in the Nucleus of HEK293 Cells: Live Cell Image Analysis

HEK293 cells that were co-transfected with the plasmids pcDNA3.1+/*HIGD2A*-GFP (HIG2A-COOH-ter coupled to green fluorescent protein) and pmCherry-C1 mCherry-NLS (red fluorescent nuclear localization signal) were used to evaluate the effect of mitochondrial stress on the subcellular localization of the HIG2A protein by live-cell confocal imaging. After 24-h of co-transfection, the HEK293 cells were exposed to mitochondrial stress for 1- or 3-h with 20 µM FCCP uncoupling agent, endoplasmic reticulum stress for 3-h with 1 µM Thapsigargin (TG), oxidative stress for 1-h with 100 µM H_2_O_2_, and chemical hypoxia for 24-h with 100 µM Cobalt (II) Chloride (CoCl_2_). FCCP alters mitochondrial proteostasis disrupting mitochondrial membrane potential [19]. The CoCl_2_-induced hypoxic condition allows us to follow HIG2A subcellular localization through live-cell imaging confocal microscopy. Z-axis slices obtained images for imaging inside the cell nucleus to observe the localization of HIG2A-GFP at the nuclear level. Green fluorescence of HIG2A-GFP colocalization in the nucleus was delimited with the red fluorescent nuclear localization signal (pmCherry-C1 mCherry-NLS). The Manders’ coefficient was assessed [20].

**Figure 4:**
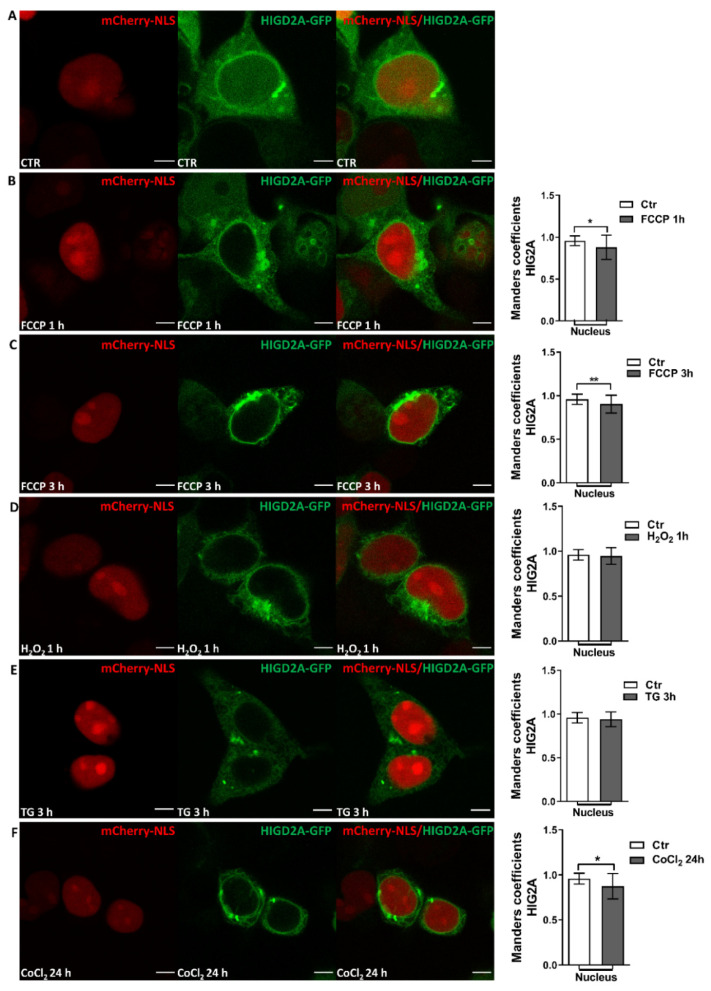
The effect of mitochondrial stress on the nuclear subcellular localization of HIG2A. The representative images of live HEK293 cells that were transfected with pcDNA3.1+/HIG2A-GFP (green) and pmCherry-C1 mCherry-NLS (red fluorescent protein with nuclear localization signal). The cell line was subjected to mitochondrial stress for 1-h and 3-h with FCCP (20 µM) (**B**,**C**); endoplasmic reticulum stress for 3-h with Thapsigargin (TG) (1 µM) (**E**); oxidative stress for 1-h with H_2_O_2_ (100 µM) (**D**), and chemical hypoxia for 24-h with Cobalt (II) (CoCl_2_) Chloride (100 µM) (**F**). The control (Ctr) (**A**) is the post-transfection basal condition. The Z-axis series (Z-axis) were obtained using confocal microscopy (Olympus FluoView FV1000 Spectral, Olympus Corp., Tokyo, Japan). The quantification of colocalization was performed using Manders’ coefficient (M2: HIG2A/Nucleus). Each bar graph represents the mean ± SE. The number of biological replicates for each condition (n) and number of cells that were analyzed in total (N°): Ctr n = 4, N° = 23 (**A**); FCCP 1 h n = 3, N° = 22 (**B**); FCCP 3 h n = 4, N° = 27 (**C**); H_2_O_2_ n = 3, N° = 28 (**D**); TG n = 3, N° = 20 (**E**); CoCl_2_ n = 2, N° = 19 (**F**), were analyzed by a one-tailed *t*-test (*p* < 0.05), followed by a Mann-Whitney test. Statistical differences were found with a significance of *p*-value (* *p*-value ≤ 0.0106 (**B**), ** *p*-value ≤ 0.0061 (**C**), * *p*-value ≤ 0.0142 (**F**)). * Significant (0.01 to 0.05). ** Very significant (0.001 to 0.01). The white bars indicate a 5 µm scale.

HIG2A-GFP colocalized with the nucleus in the control condition (Figure 4A). Upon treatment with the mitochondrial uncoupler, FCCP for 1-h and 3-h, the HIG2A protein decreased its colocalization at the nucleus. Also, FCCP increased the HIG2A-GFP perinuclear distribution (Figure 4B,C and Figure S3, and Table 3).

The treatment with H_2_O_2_ and TG produced no changes in the distribution of HIG2A at the nucleus in HEK293 cells (Figure 4D,E and Table 3).

The above is related to the increase of HIG2A fluorescence in the mitochondrial network. The HIG2A protein decreased its colocalization in the nucleus when the HEK293 cells were subjected to mitochondrial (FCCP) and extreme chemical hypoxic (CoCl2) stress, and the HIG2A protein localization was shifted to the mitochondria (Figure 4F, and Table 3).

## 3. Discussion

Mitochondria have their mitochondrial genome (mtDNA) consisting of a circular molecule of 16.5 Kb that is essential for OXPHOS [21]. The mtDNA codes only for 13 out of 1200 proteins that are described for mitochondrial biology [21]. This situation adds greater complexity to the organization, transcription, translation, and translocation of mitochondrial proteins that are continuously coordinated between the nucleus and mitochondria [21]. The coordination mechanisms include signals from the mitochondria to the nucleus (retrograde communication) and from the nucleus to the mitochondria (anterograde communication) [22]. Anterograde signaling coordinates gene expression in the mitochondria in response to endogenous or environmental stimuli that are detected by the nucleus. In contrast, retrograde signaling transmits signals originating in the mitochondria to regulate nuclear gene expression [22].

The retrograde communication pathway is induced by mitochondrial dysfunction such as loss of mitochondrial DNA, a decrease in the synthesis of mitochondrial ATP, an increase in mitochondrial ROS levels, or mitochondrial membrane depolarization. Thus, activating the retrograde signaling pathway requires a sensor of mitochondrial dysfunction and the transcription factors, which leads to the induction of the stress response genes, metabolic energy damage, and proteo-toxic stress or OXPHOS mitochondrial biogenesis [22,23,24,25,26,27,28,29,30]. Posttranslational modifications might translocate the transcription factors to the nucleus. Once in the nucleus, the transcription factors bind to the retrograde response target genes [22,25,26,27,28,29,30,31,32].

**Figure 5:**
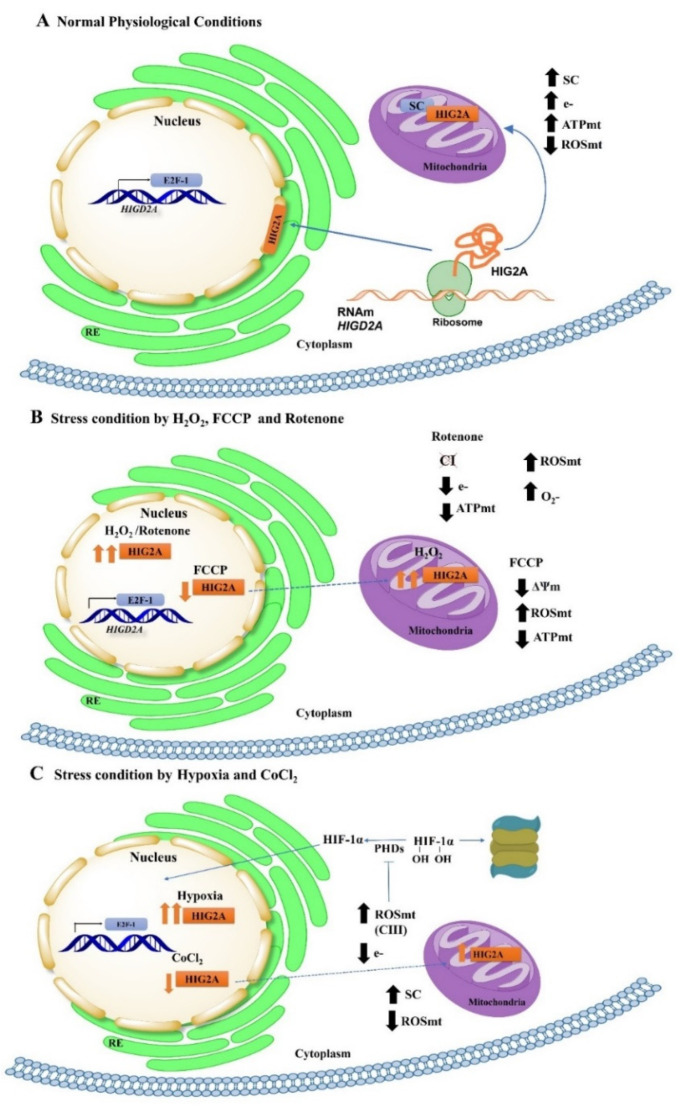
Model of HIG2A behavior in response to stress. (**A**) The transcription factor E2F-1 controls the HIGD2A gene, which encodes the HIG2A protein (in orange) [12]. The model describes two “pools” of HIG2A protein. Once synthesized in the cytoplasm, the HIG2A protein is distributed at the mitochondria and the nucleus [12,17]. Currently, the function of HIG2A at the nucleus has not been described, whereas, at mitochondria, HIG2A is localized in the inner mitochondrial membrane where it participates in the formation of respiratory supercomplexes (SC), interacting with complex III (CIII) and complex IV (CIV), and promoting their stability [8,11,12]. The increase in the formation of respiratory supercomplexes has been associated with a decrease in mitochondrial reactive oxygen species (ROSmt) and an increase in mitochondrial energy generation (ATPmt) [1,2,4,6]. The distribution of HIG2A protein at the subcellular level will depend on the type of stress to which cells are exposed. (**B**) Mitochondrial stress by Rotenone (Complex I (CI) inhibitor) causes an increase of HIG2A at the nuclear level. Inhibition at the CI level causes an increase in superoxide anion (O^2-^) generation and a decrease in electron (e-) flow through the electron transport chain (ETC) and mtATP generation [67,68]. In contrast, FCCP causes a reduction at the nucleus, which could suggest (dotted arrow) a shift of HIG2A into the mitochondria to make up for the deficiency in mitochondrial membrane potential (ΔΨm) that is caused by the mitochondrial uncoupler FCCP [69], participating in SC formation, stabilizing electron flow, and maintaining mitochondrial energy generation (mtATP). In comparison, H_2_O_2_ (generalized stress) caused an increase in HIG2A at the mitochondria and cytoplasm. H_2_O_2_ causes an increase in ROS at the cellular level which may participate in signaling pathways. (**C**) It has been described that under hypoxic conditions, the transcription factor HIF-1α is stabilized and forms a heterodimer with the transcription factor HIF-1β recognizing consensus sequences of the target genes that are involved in lactic acid formation [70]. At mitochondria, mtROS that are generated by complex III (CIII) can inhibit the enzyme prolyl hydroxylase (PHD), which hydroxylases (OH) the transcription factor HIF-1α, for subsequent degradation via the proteasome [71]. In addition, the decrease in oxygen (O_2_) levels causes an increase in the formation of SCs, which helps prevent electrons from escaping, thus decreasing mtROS generation [71,72,73]. The subcellular distribution of HIG2A by hypoxic stress that is caused by physical hypoxia of 2% O_2_ and by chemical hypoxia by CoCl_2_ (hypoxia equal to or less than 1% O_2_) causes the HIG2A protein to present a differential distribution. Hypoxia of 2% O_2_ causes an increase of HIG2A at the nucleus, which could indicate new functions that are not yet described at the nuclear level and which could be regulated by stress factors. Conversely, CoCl_2_ stress causes a decrease in HIG2A at the nucleus. This decrease could indicate (dotted arrow) a shift of HIG2A to the mitochondrial level to promote SC formation and stability, decrease mtROS generation, and provide more efficient electron transport through the mitochondrial transport chain.

HIG2A nuclear localization is related to the stress response [12,17,33], suggesting that HIG2A can participate in the communication processes between the mitochondria and the nucleus. Mitochondrial oxidative stress with rotenone increases HIG2A protein levels at the nucleus (Figure 1A). Whereas generalized oxidative stress with H_2_O_2_ increases HIG2A at the nucleus and cytoplasm (Figure 1B), suggesting that the subcellular distribution of HIG2A protein depends on the type of oxidative stress to which the cells are exposed (Figure 5). In contrast, exogenous HIG2A-GFP protein decreases the nuclear colocalization in HEK293 cells that are treated with FCCP (Figure 4). This decrease could be associated with the translocation of HIG2A-GFP to other subcellular compartments, such as the mitochondrial network (Figure 4).

In HEK293 and C2C12 cell lines, HIG2A colocalization is higher at the nucleus compared with the mitochondria in both oxygen conditions (Figure 2 and Figure 3). Hypoxic stress (2% O_2_) for 24- and 48-h does not alter the distribution of HIG2A in HEK293 cells (Figure 2). While in C2C12 cells, HIG2A increases colocalization in the nucleus during 48-h hypoxia versus normoxia (Figure 3B). This suggests that hypoxic stress increases the nuclear localization of HIG2A in C2C12 cells, which could be related to cell type and metabolism. In contrast, hypoxic chemical stress with CoCl_2_ decreases HIG2A-GFP nuclear colocalization in the HEK293 cells (Figure 4). These differences may be related to the differences in oxygen concentration. It is considered that chemical hypoxia with CoCl_2_ can reach less than 1% oxygen [34]. This could indicate that HIG2A is a protein that is sensitive to low oxygen concentrations and its distribution pattern at the nucleus changes during a prolonged (toxic) chemical hypoxia.

HIG2A expression is stimulated by the hypoxia-inducible factor 1 (HIF-1), and their over-expression promotes cell survival in different models [8,9,10,12,13,35,36,37]. In HEK293 cells, induction is observed between 24- to 48-h of hypoxia and in C2C12 between 36- to 48-h of hypoxia [12]. In addition, the HIG2A protein level increased in pathological hypoxia (1% O_2_) during the first few hours of exposure [11]. HIG2A could function as a hypoxia sensor in respiratory supercomplexes to activate signaling pathways of response to hypoxic stress. In the literature, there is evidence of other proteins having similar behavior. That is the case of STAT3 which functions in the nucleus as a transcription factor [38,39,40] and in mitochondria to promote energy homeostasis under stress conditions [41,42]. STAT3 activation by hypoxia (1% O_2_) produces its translocation to the nucleus in HeLa cells and human endothelial cells (EA.hy926). Intriguingly, in HeLa, STAT3 translocation is higher at 24-h, while in EA.hy926, it translocates after 6-h of hypoxia (1% O_2_) [43]. In comparison, chemical hypoxia with the iron chelator deferoxamine inhibits STAT3 translocation to the nucleus in both cell lines [43].

The translocation of proteins between the mitochondria and the nucleus is mainly mediated by stress. For example, the mitochondrial ORF of the 12S rRNA-c (MOTS-c) peptide that are encoded by mtDNA regulates a wide range of genes in response to stress. MOST-c is translocated to the nucleus in response to metabolic stress (glucose restriction, serum deprivation, and oxidative stress) that triggers ROS generation. Conversely, treatment with N-acetylcysteine inhibits MOST-c translocation to the nucleus [44,45].

The G-protein pathway suppressor 2 (GPS2) has a dual mitochondrial/nuclear localization and regulates mitochondrial function through transcriptional regulation of mitochondrial genes that are encoded in the nucleus [30]. Mitochondrial membrane depolarization induces GPS2 translocation from the mitochondria to the nucleus and promotes the transcriptional activation of the mitochondrial stress response genes that are encoded in the nucleus [30]. GPS2 subcellular redistribution is associated with a sumoylation post-translational modification in the mitochondria. The SUMO protease SENP1 mediates the desumoylation regulating the GPS2 retrograde translocation [30].

Mitochondrial-nuclear communication is one of the components that is required for mitochondrial unfolded protein response (mtUPR) [46]. When mitochondria accumulate misfolded proteins or an imbalance in the stoichiometry of the mitochondrial respiratory chain due to stress, the mitochondria sense the damage, activating a nuclear transcription factor, which expresses a series of proteases and chaperones that repair the damage that is sensed [47,48,49]. In *Caenorhabditis elegans*, the transcription factor ATFS-1 possesses dual subcellular localization sequences after cellular stress, which allow mediating the mitochondrial-nuclear communication [50]. The mitochondrial import efficiency of ATFS1 regulates the mtUPR in *C. elegans*. Once ATFS1 is imported into the mitochondrial matrix, it is degraded. With mitochondrial dysfunction, ATFS1 cannot reach the mitochondrial matrix and instead translocates to the nucleus where it activates nuclear-encoded genes such as mitochondrial chaperones and proteases, ROS detoxification enzymes, and mitochondrial protein import components [46,50,51]. In mammals, it has been reported that the CEBP homologous protein (CHOP), the activating transcription factor 4 (ATF4), and the activating transcription factor 5 (ATF5) are orthologous of ATFS1 [46]. Recently, it was described that mitochondrial stress response in mammals activates the transcription factor ATF4 [19]. ATF5 presents a mitochondrial import efficiency, similar to ATFS1 [46]. CHOP and CCAAT/enhancer-binding protein (CEBP-β) form a heterodimer that is involved in activating mitochondrial chaperones and proteases (mtUPR). The heterodimer CHOP/CEBP-β fulfills the function most similar to ATFS-1 [48].

Our current investigation elucidates that HIG2A responds to mitochondrial stress that is related to nuclear localization. Although, we still need further experimental work to verify if its dual presence between the mitochondria and the nucleus is related to a role in the coordinated communication between the nucleus and the mitochondria. Also, we could speculate that the synthesis of HIG2A protein at the ribosomal level in the cytoplasm could generate two pools of HIG2A protein, one that is distributed to the nucleus with functions that are not yet described, and another at the mitochondrial level where it participates in respiratory supercomplexes. The subcellular distribution between both organelles could be strongly related to the type of stress (Figure 5). It is essential to know which amino acid residues enable localization in both organelles to understand what allows HIG2A subcellular localization. We expect the likely non-canonical importin α-dependent nuclear localization signal (NLS) of HIG2A [17] to be indispensable for translocation to the nucleus. Besides, HIG2A has a putative motif of DNA-binding residues in the alpha-helix [17]. Moreover, HIG2A has a probable SUMO interaction motif and a SUMOylating nonconsensus residue [17]. The sumoylation could regulate the nuclear localization of some proteins [30].

The mechanisms that are involved in communication between mitochondria and the nucleus are an emerging topic of study. Progress is required for understanding the critical mediators that are involved in the signaling pathways between the two organelles [52]. Questions arise regarding how factors that are involved in the communication pathways between the two organelles move through the cytosol and how this movement is regulated. It has been proposed that the mobility of mitochondria toward a perinuclear cluster may be required, possibly through the formation of contact sites between the nucleus and mitochondria similar those that are described at the junctions between the mitochondrial membranes with the endoplasmic reticulum (ER) [52]. This could begin to partially explain the presence of HIG2A in the nuclear membrane [12,17].

The HIG2A protein is homologous to the HIG1A protein; both are members of the hypoxia-inducible domain family. Ameri et al. (2013) reported that in hypoxia (2% O_2_), endogenous HIG1A localized mainly in the mitochondria. When subjected to ischemia (1% O_2_ or together with glucose starvation) or with the DNA damaging agent etoposide, endogenous HIG1A was observed localizing at the nucleus [33]. In our results, ROS (O_2−_ and H_2_O_2_) stress, the leading cause of DNA damage, favors the presence of HIG2A at the nucleus (Figure 1). Also, HIG2A significantly increases colocalization in the nucleus during hypoxia in C2C12 cells (Figure 3).

Timón-Gómez et al.’s (2020) results suggested that HIG1A and HIG2A proteins are not detected in the nuclear fraction from HEK293T cells [11]. The HEK293T cell line is derived from HEK293. HEK293T stably expresses the SV40 (polyomavirus) Large T antigen. This antigen alters gene expression and host cell growth by binding to transcription factors, components of the replication machinery, and cell cycle regulatory apparatus, including p53 and retinoblastoma family proteins such as pRb [53,54]. Most T antigen interactions with these cellular proteins are crucial for tumorigenesis [53,55]. *HIGD2A* gene is regulated by the transcription factor E2F-1, which is regulated by the pRb protein [12]. SV40 Large T antigen could be influencing the localization of HIG1A and HIG2A proteins in the nucleus.

Lately, we have performed a biosystem analysis of the HIG2A and its implications in cancer biology. The analysis indicates that the *HIGD2A* gene is not implicated in cancer via mutation. In addition, DNA methylation and mRNA expression of the *HIGD2A* gene display significant alterations in several cancers. Some cancers with a high expression of *HIGD2A* present a downward trend survival of patients [17]. We suggest that HIG2A may play a role in reprogramming the metabolism. In cancer, metabolic reprogramming is mainly promoted by the overexpression of oncogenes and the loss of function of tumor suppressor genes [56]. Recently, it has been established that mitochondrial metabolism is involved in transformation, tumor progression, cancer cell proliferation, and the acquisition of resistance by tumor cells to a hostile environment [57]. One of the main mechanisms that are used by mitochondria for cancer cell transformation was found to be ROS production, which favors the accumulation of potential oncogenic defects in DNA [58]. The contribution of mitochondrial respiration in oncogeneses is studied in various types of cancer that exhibit elevated levels of electron transport chain proteins (complexes I, III, and IV) that remodel respiratory supercomplexes during tumorigenesis and metastasis [59,60]. This oxidative metabolism is shown in malignant hepatocellular carcinoma [61], colorectal cancer [62], clear cell renal carcinoma [62], breast cancer [63,64], diffuse large B-cell lymphomas [65], melanoma [60], and classical Hodgkin’s lymphoma [66].

## 4. Materials and Methods

### 4.1. Cell Lines

HEK293 cells were grown in Dulbecco’s Modification of Eagle’s Medium (DMEM) with 4.5 g/L glucose, L-glutamine, and sodium pyruvate (10013CVR-Corning, Corning Incorporated, Life Sciences, Tewksbury, MA, USA) that was supplemented with 15 mM HEPES, 100 U/mL Penicillin-Streptomycin, 2 mM L-glutamine, and 10% Fetal Bovine Serum (FBS). C2C12 was grown in DMEM with 4.5 g/L glucose, L-glutamine, and sodium pyruvate (10013CVR-Corning) that was supplemented with 15 mM HEPES, 100 U/mL Penicillin-Streptomycin, 2 mM L-glutamine, and 20% FBS.

### 4.2. Hypoxia Induction

The cells were subjected to normal oxygen conditions or normoxia (21% O_2_) and hypoxic stress (2% O_2_). In both conditions, the cells were grown in DMEM with glucose, L-glutamine, and sodium pyruvate (10013CVR-Corning), that was supplemented with 6 mM bicarbonate, 25 mM HEPES, 100 U/mL Penicillin-Streptomycin, 2 mM L-Glutamine, and 20% FBS for C2C12 cells and 10% for HEK293 cells was used. The hypoxia levels that range from 0.5% to 2% oxygen are considered pathological hypoxia [74]. Hypoxia was performed using a hypoxia chamber (Biospherix, Parish, NY, USA) within the CO2 incubator (37 °C, 5% CO_2_), the oxygen level was regulated by ProOx 110 (Biospherix) [12].

### 4.3. Induction of Cellular and Mitochondrial Stress by Pharmacological Treatments

We used the HEK293 cell line to perform oxidative stress induction assays. The cells were grown on glass, and once they reached 60% confluence, they were treated with 100 µM H_2_O_2_ [75,76] or with 20 nM rotenone for 1 h [67,68,77]. Then, the cells were fixed for immunofluorescence according to the protocol that is detailed in “Section 4.4”.

For the assessment of HIG2A localization with live-cell imaging confocal microscopy, the HEK293 cells were lipofectamine-co-transfected with the plasmids pcDNA3.1+/*HIGD2A*-GFP (*HIGD2A* [NM_138820] ORF Clone Catalog OHU31187D GenScript Biotech, Piscataway, NJ, USA) and pmCherry-C1 mCherry-NLS (pmCherry-C1 mCherry-NLS was a gift from Dyche Mullins [Addgene Plasmid #58476] Addgene, Watertown, MA, USA) [78]). One day after transfection, the cells were treated for 1- or 3-h with 20 µM FCCP (carbonylcyanide-4-(trifluoromethoxy) phenylhydrazone [69], 100 µM H_2_O_2_ for 1-h, 3-h with 1 µM TG (Thapsigargin), and 100 µM CoCl_2_ for 24-h. FCCP (20 µM) for 1-h and 3-h induced mitochondrial stress; this treatment was based on the following study. FCCP [10 µM] causes a rapid decline in mitochondrial membrane potential (∆ ψm) as early as 15-min after treatment [69]. The selection of H_2_O_2_ (100 µM) for 1-h treatment was based on the following studies. Short exposure to H_2_O_2_ (100 µM) for 1-h in the HEI-OC1 cells induces changes in mitochondria morphology, mitochondrial depolarization, decreased O_2_ consumption rate, and the mPTP opening [75]. H_2_O_2_ is freely diffusible, causing oxidation in other cellular compartments beyond the mitochondria. The treatment of HEK293 with 100 μM H_2_O_2_ for 20-min served as the positive control of singlet oxygen-induced mitochondrial dysfunction [76]. Finally, rotenone (20 nM) for 1-h was based on the following studies. Rotenone 5 nM increases mitochondrial superoxide (O_2−_) levels and potentiates glutamate-induced cytoplasmic Ca^2+^ deregulation. Rotenone at 20 nM inhibited basal and FCCP-stimulated cell respiration and caused respiratory failure in the presence of glutamate [67]. Chronic exposure to rotenone 20–30 nM cause degeneration of dopaminergic neurons in the midbrain [68]. Rotenone (10 nM) decreased intracellular ATP levels and ΔΨm in real-time change [77].

### 4.4. Indirect Immunofluorescence (IFA)

To evaluate the effect of hypoxic stress on the subcellular localization of the HIG2A protein, we used the HEK293 and C2C12 cell lines. Totals of 4 × 10^4^ cells were plated on 35 mm × 10 mm plates with circular coverslips of 16 mm diameter (Knittel glass, Brunswick, Germany) that were treated with Poly-L-Lysine (Sigma-Aldrich, ST. Lois, MO, USA). Once the cell cultures reached adequate confluence under normoxia 21% O_2_, they were subjected to hypoxic stress (2% O_2_) for 24- or 48-h [12,79]. Once the hypoxic stress was terminated, the mitochondria were labeled with either the MitoTracker^TM^ Red CM-H2XRos probe (Invitrogen^TM^, ThermoFisher Scientific, Waltham, MA, USA) (red fluorescent staining) or the MitoTracker^TM^ Deep Red FM probe (Invitrogen^TM^, ThermoFisher Scientific, Waltham, MA, USA) (fluorescent staining in the far red). Both probes stain mitochondria in living cells and their accumulation is dependent on membrane potential. Incubation with the probe was for 30-min at 37 °C. The cells were then washed with 1X DPBS that was supplemented with 0.1 mM CaCl_2_ and 1 mM MgCl_2_ and fixed for 7 min with 4% paraformaldehyde at room temperature. The paraformaldehyde was removed with three washes of 3 min each with 1X DPBS that was supplemented with 0.1 mM CaCl_2_ and 1 mM MgCl_2_. Then, the cell membrane was permeabilized with 0.1% Triton X-100 (Sigma-Aldrich) for 2 min and subsequently washed three times for 5 min each wash with 1X DPBS that was supplemented with 0.1 mM CaCl_2_ and 1 mM MgCl_2_. The cells were incubated with 1% BSA, 0.3 M glycine in PBST (PBS + 0.1% Tween20) for 30 min at room temperature to block possible nonspecific binding. This was followed by three washes of 5 min each with 1X DPBS that was supplemented with 0.1 mM CaCl_2_ and 1 mM MgCl_2_ and incubated with the primary antibody that was specific for HIG2A protein (1:500) for human cells (Abcam, ab135399, Cambridge, UK) or mouse cells (Abcam, ab150893). The primary antibodies were diluted in 1% BSA in PBST and incubated in a humidified chamber overnight at 4 °C. Next, coverslips were washed three times in the dark with 1X DPBS that was supplemented with 0.1 mM CaCl_2_ and 1 mM MgCl_2_ and incubated with the secondary antibody DyLightR 488 (green) diluted in 1% BSA in PBST for 1-h at room temperature. 1 μg/mL Hoescht (Sigma-Aldrich, ST. Lois, MO, USA) in PBS for 2-min at room temperature was used as a fluorescent nuclear marker. Finally, the excess Hoescht was removed and washed with PBS1X. The coverslip was mounted on a slide on a drop of Fluoromount-G™ Mounting Medium (ThermoFisher Scientific, Waltham, MA, USA). Once dried, it was permanently sealed with enamel and stored at 4 °C in the dark for further analysis with epifluorescence microscopy or confocal microscopy [17]. In each immunofluorescence experiment, the corresponding fluorescent secondary antibody control was performed, omitting the primary antibody to determine the specificity of the secondary antibody signal. The levels of HIG2A colocalization at the mitochondrial and nuclear levels were analyzed by analysis of the images that were obtained by confocal microscopy (Leica TCS SP8, Leica Microsystems Inc., Mannheim, Germany. Version: 3.4.218368).

### 4.5. Image Acquisition and Processing

There were two confocal microscopes that were used for the colocalization study: an Olympus Fluoview FV 1000 (Olympus Corp., Tokyo, Japan. Version: 04.02.03.06) and a Leica TCS SP8 (Leica Microsystems Inc., Mannheim, Germany. Version: 3.4.218368). Immunofluorescence acquisition was performed on a Leica TCS SP8 confocal microscope with HC PL APO CS2 63X oil optical magnification, Numerical Aperture (NA) 1.4, and 5X digital zoom. Confocal micrographs were acquired using excitation wavelengths of 405 nm (Hoescht), 488 nm (DyLight^®^ 488), and 552 nm (MitoTracker^TM^ Red). A total of three spectral detectors (PMT) detected the signals; PMT1 (410–480 nm) Hoescht, PMT2 (495–547 nm) DyLight^®^ 488, and PMT3 (560–620 nm) MitoTracker^TM^ Red. Image acquisition was performed on a Leica TCS SP8 confocal microscope with 63X optical magnification and 5X digital zoom. The images were acquired with 22 Z-axis sections with 0.130 microns separation between each slice (Z step size) and saved in LIF format (Leica microsystems). The images were deconvoluted using LASX software version for Life Science (AutoQuant Deconvolution Algorithms licensed from Media Cybernetics Inc. Copyright 2009, Mannheim, Germany).

Olympus FluoView FV1000 Spectral confocal microscope with a cell incubator system (Live Cell Instrument, Chamlide IC, Quorum Technologies, Seoul, Korea) acquired live-cell images using a 60X oil objective and 5X digital zoom. Confocal micrographs were acquired using excitation wavelengths of 488 nm (pcDNA3.1+/*HIGD2A*-GFP (green) and 543 nm (pmCherry-C1 mCherry-NLS).

Images were deconvoluted using Huygens software version 18.02 (Scientific Volume Imaging). A total of 15 iterations were used using the “blind Deconvolution” method (Point Spread Function—PSF). Subsequently, image processing was performed with software Fiji (Image J 1.52p) [80].

The background subtraction (Rolling Ball radius: 50.0 pixels) and the Otsu Kittler threshold were applied for image processing of each channel (HIG2A protein, mitochondrion, and nucleus) [81]. Binary masks were made for channels identification in the ROI Manager. The plugin, Coloc2, was used to perform colocalization studies [80].

As appropriate, the binary mask of nucleus or mitochondrion is used to analyze colocalization with HIG2A protein. Costes significance test was with a PSF of 3.0. [82]. A total of 50 Costes randomizations were used to obtain Manders’ coefficients, which measures the fraction of one structure’s pixels that colocalize with the other structure [20].

Colocalization degrees were assigned as described in Zinchuk et al. [83]. The set includes Manders’ description for five variables: 0~0.54 “Very weak”, 0.55~0.77 “Weak”, 0.78~0.94 “Moderate”, 0.96~0.98 “Strong”, and 0.99~1.0 “Very strong”, which was used as a standard to describe the results of quantitative colocalization studies [83].

The HIG2A intensity fluorescence analysis images were acquired using an epifluorescence microscope with an LED illumination system and filters to detect four independent fluorescence channels (Leica DMI6000). Quantifying HIG2A fluorescence at the cellular and nuclear levels were performed using binary masks through the particle analysis of the ImageJ software. With the ROI manager the whole cell and the nucleus was identified. A cytoplasm fluorescence value was obtained by subtracting nucleus fluorescence from cell fluorescence.

### 4.6. Statistical Analysis

All statistical analyses were performed with GraphPad Prism 6 software (Prism Windows 6.07, GraphPad, San Diego, CA, USA). An unpaired Student’s *t*-test followed by a Mann-Whitney test was used when comparing two mean values. A one-way ANOVA followed by a Dunnett’s multiple comparison test was also performed. All analyses with *p* ≤ 0.05 were considered statistically significant. The data represent the mean ± standard error of the mean (SEM).

## 5. Conclusions

HIG2A protein levels are increased by cellular stress such as hypoxia and oxidative stress. Moreover, HIG2A protein shows a dynamic distribution between the mitochondria and the nucleus in response to stress factors. Interestingly, HIG2A protein colocalizes at a higher level in the nucleus compared with the mitochondrial network under normoxia and hypoxia. Although, HIG2A increases its mitochondrial localization in chemical hypoxia (anoxia o severe hypoxia).

Increased mRNA levels of the *HIGD2A* gene may be related to oncogenic features that could enhance cancer cell proliferation, which would explain the trend of low survival probability in cancer patients with increased *HIGD2A* gene expression [17]. Experimental verification of the effects of overexpression or deletion of the *HIGD2A* gene on cancer cell proliferation is required. The dynamic distribution of HIG2A between the mitochondria and the nucleus in response to stress factors may represent a new communication system between the mitochondria and the nucleus. One possible translational medicine application of the findings in this study is targeting HIG2A in cancer therapy to silence *HIGD2A* and induce death in cancer cells.

## Figures and Tables

**Table 1:** The effect of hypoxic stress on the subcellular localization of HIG2A in HEK293.

Treatment	HIG2A/Nucleus [M2]	HIG2A/Mitochondria [M1]	*p*-Value	Nucleus/HIG2A [M1]	Mitochondria/HIG2A [M2]
Normoxia 24 h	0.95	0.85	** *p* ≤ 0.0011	0.84	0.90
Hypoxia 24 h	0.94	0.88	** *p* ≤ 0.0011	0.79	0.95
Normoxia 48 h	0.96	0.78	*** *p* ≤ 0.0001	0.89	0.95
Hypoxia 48 h	0.94	0.82	** *p* ≤ 0.0029	0.85	0.93

** Very significant (0.001 to 0.01). *** Extremely significant (0.0001 to 0.001). Colocalization degrees for Manders’: 0~0.54 “Very weak”, 0.55~0.77 “Weak”, 0.78~0.94 “Moderate”, 0.96~0.98 “Strong”, and 0.99~1.0 “Very strong”. Manders’ coefficient (M1 and M2), measuring the fraction of pixels in one structure overlapping with the pixels in other structures.

**Table 2:** Effect of hypoxic stress on the subcellular localization of HIG2A in C2C12.

Treatment	HIG2A/Nucleus [M2]	HIG2A/Mitochondria [M1]	*p*-Value	Nucleus/HIG2A [M1]	Mitochondria/HIG2A [M2]
Normoxia 24 h	0.98	0.88	*** *p* ≤ 0.0001	0.94	0.89 ^2^
Hypoxia 24 h	0.97	0.86	** *p* ≤ 0.0073	0.97	0.94 ^2^
Normoxia 48 h	0.99	0.83	*** *p* ≤ 0.0001	0.86 ^1^	0.85 ^3^
Hypoxia 48 h	0.97	0.82	*** *p* ≤ 0.0002	0.97 ^1^	0.97 ^3^

** Very significant (0.001 to 0.01). *** Extremely significant (0.0001 to 0.001). ^1^ Nucleus/HIG2A (M1) at Normoxia 48 h versus Nucleus/HIG2A (M1) at Hipoxia 48h ***, *p*-value 0.0004; ^2^ Mitochondria/HIG2A (M1) at Normoxia 24 h versus Mitochondria/HIG2A (M1) at Hipoxia 24 h ***, *p* ≤ 0.0002; ^3^ Mitochondria/HIG2A (M1) at Normoxia 48 h versus Mitochondria/HIG2A (M1) at Hipoxia 48 h ***, *p* ≤ 0.0001. Colocalization degrees for Manders’: 0~0.54 “Very weak”, 0.55~0.77 “Weak”, 0.78~0.94 “Moderate”, 0.96~0.98 “Strong”, and 0.99~1.0 “Very strong”. Manders’ coefficient (M1 and M2), measuring the fraction of pixels in one structure overlapping with the pixels in other structures.

**Table 3:** The effect of mitochondrial stress on the subcellular localization of HIG2A in the nucleus of HEK293 cells: live-cell image analysis.

Treatment	HIG2A/Nucleus [M2]	*p*-Value
Control	0.95	
FCCP 1 h	0.88	* *p*-value ≤ 0.0106
FCCP 3 h	0.90	** *p*-value ≤ 0.0061
H_2_O_2_ 1 h	0.95	
TG	0.94	
CoCl_2_	0.87	* *p*-value ≤ 0.0142

* Significant (0.01 to 0.05). ** Very significant (0.001 to 0.01). Colocalization degrees for Manders’: 0~0.54 “Very weak”, 0.55~0.77 “Weak”, 0.78~0.94 “Moderate”, 0.96~0.98 “Strong”, and 0.99~1.0 “Very strong”. Manders´ coefficient (M2), measuring the fraction of pixels in one structure overlapping with the pixels in other structures.

## Data Availability

Not applicable.

## Supplementary Materials

The following supporting information can be downloaded at https://www.mdpi.com/article/10.3390/ijms23010389/s1.
