# Supplementary material for: Dynamic Distribution of HIG2A between the Mitochondria and the Nucleus in Response to Hypoxia and Oxidative Stress
Source: Int J Mol Sci. 2021 Dec 30;23(1):389. doi: 10.3390/ijms23010389 (PMC8745331; doi:10.3390/ijms23010389)
Supplement: Supplementary file 1 [file ijms-23-00389-s001.zip › ijms-1463654-supplementary.pdf]

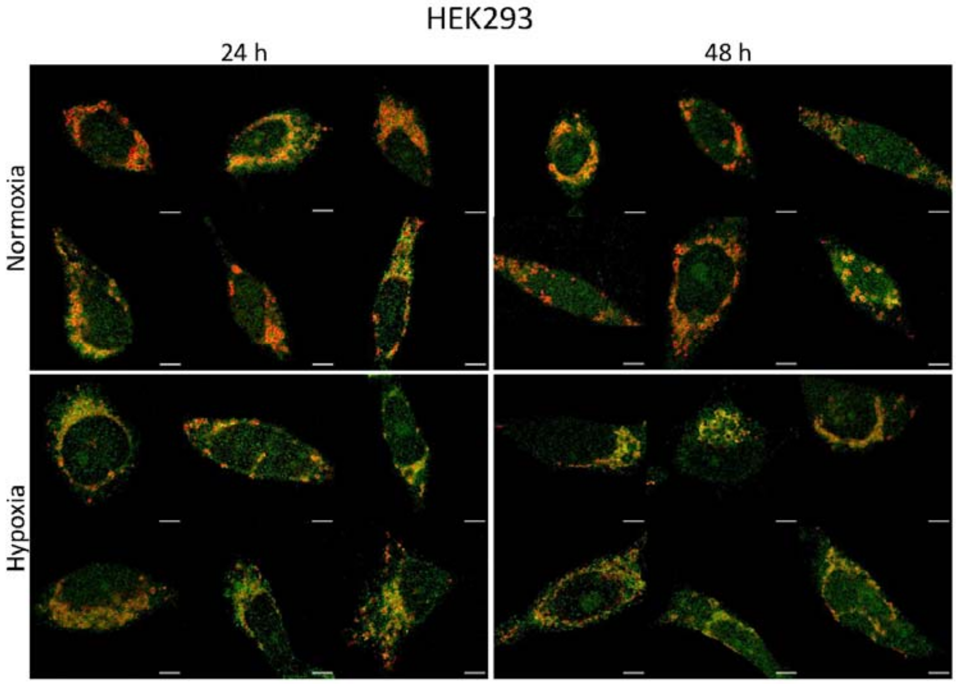

Figure S1. Effect of hypoxia on subcellular localization of HIG2A in mitochondria and nucleus of HEK293 cells. Representative immunofluorescence images in HEK293 cells; Anti-HIG2A primary antibody (Abcam ab135399), DyLight® 488 secondary antibody (HIG2A green). MitoTracker® Red CM-H2XRos mitochondrial fluorescent marker (M red). Hoescht 33342 (blue signal - DNA). Z-axis series were obtained using confocal microscopy (Leica SP8). The separation between each of the slices (Z step size) was 0.130 microns. HEK293 cells were subjected to hypoxic stress (2% O_2) for 24Hrs and 48Hrs.

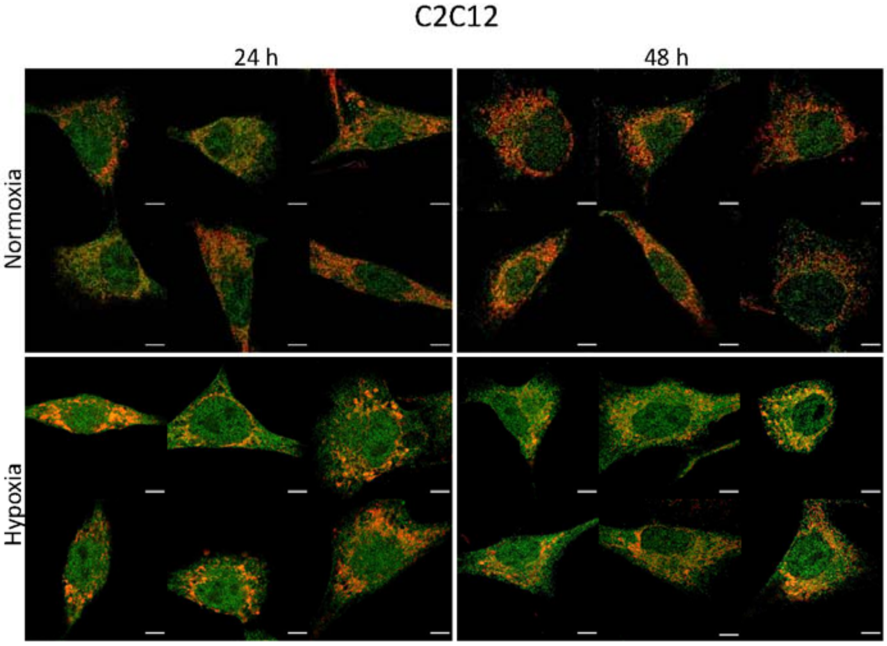

Figure S2. Effect of hypoxia on subcellular localization of HIG2A in mitochondria and nucleus of C2C12 cells. Representative immunofluorescence images in C2C12 cells; Anti-HIG2A primary antibody (Abcam ab135399), DyLight® 488 secondary antibody (HIG2A green). MitoTracker® Red CM-H2XRos mitochondrial fluorescent marker (M red). Hoescht 33342 (blue signal - DNA). Z-axis series were obtained using confocal microscopy (Leica SP8). The separation between each of the slices (Z step size) was 0.130 microns. C2C12 cells were subjected to hypoxic stress (2% O_2) for 24 h and 48 h.

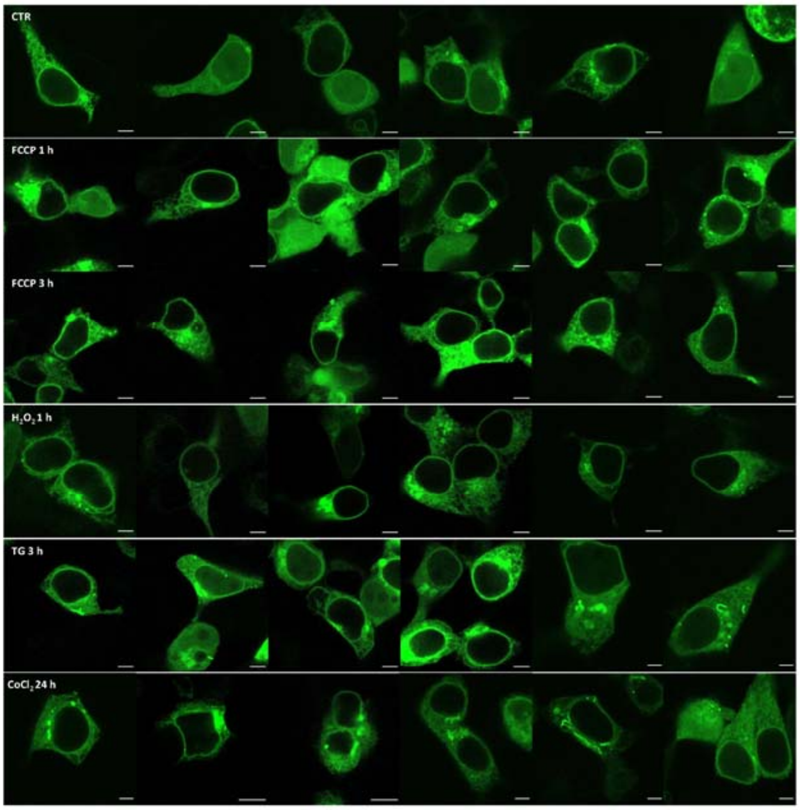

Figure S3. Effect of mitochondrial stress on nuclear subcellular localization of HIG2A. Representative images of live HEK293 cells transfected with pcDNA3.1+/HIG2A-GFP (green) and pmCherry-C1 mCherry-NLS (red fluorescent protein with nuclear localization signal). The cell line was subjected to mitochondrial stress for 1hr and 3hrs with FCCP (20 μM); endoplasmic reticulum stress for 3 h with Thapsigargin (TG) (1 μM); oxidative stress for 1hr with H_2O_2 (100 μM) and chemical hypoxia for 24 h with Cobalt (II)($CoCl_2$) Chloride (100μM). The control (Ctr) is the post-transfection basal condition. Z-axis series (Z-axis) were obtained using confocal microscopy (Olympus FluoView FV1000 Spectral).
